# Time course transcriptome data analysis for in vitro modeling of dilated cardiomyopathy using patient-derived induced pluripotent stem cells

**DOI:** 10.1186/1471-2105-16-S15-P8

**Published:** 2015-10-23

**Authors:** Xing Li, Saranya Wyles, Sybil C Hrstka, Jean-Pierre A Kocher, Andre Terzic, Timothy M Olson, Timothy J Nelson

**Affiliations:** 1 Division of Biomedical Statistics and Information, Department of Health Sciences Research, Mayo Clinic College of Medicine, Rochester, MN 55905, USA; 2 Center for Clinical and Translational Sciences, Mayo Clinic College of Medicine, Rochester, MN 55905, USA; 3 Division of General Internal Medicine Mayo Clinic College of Medicine, Rochester, MN 55905, USA; 4 Marriott Heart Disease Research Program, Division of Cardiovascular Diseases, Mayo Clinic College of Medicine, Rochester, MN 55905, USA; 5 Department of Molecular Pharmacology and Experimental Therapeutics, Mayo Clinic College of Medicine, Rochester, MN 55905, USA; 6 Division of Pediatric Cardiology, Mayo Clinic College of Medicine, Rochester, MN 55905, USA

## Background

Induced pluripotent stem cells (iPSCs) derived from dilated cardiomyopathy (DCM) patients offer an unprecedented platform for in vitro disease modeling[1]. Time course transcriptome analysis on the differentiation process from iPSCs to beating cardiomyocytes will reveal the holistic dynamic gene expression landscapes and pinpoint molecular deficiencies in cardiogenesis for DCM patients.

## Materials and methods

In this study, dermal fibroblasts were isolated from skin biopsies of two unrelated patients who carry the RBM20 R636S mutation. The dermal fibroblasts were reprogrammed to iPSCs and then differentiated to cardiomyocytes to model the cardiogenesis for DCM patients. During the differentiation process, Cell samples at five stages (day 0, 10, 15, 20, and 25) were collected and RNA was extracted for time course transcriptome analysis. The iPSCs from a healthy subject was used as control.

## Results

Unsupervised hierarchical clustering on genome-wide expression profiles defined clearly separated developmental stages containing pluripotent samples (day 0), early cardiac samples (day 10 and 15), and late cardiac samples (day 20 and 25). Furthermore, Principal Component Analysis revealed dramatic transcriptome differences on patients with severe and minor phenotypes. The comparison of transcriptome profiles of two *RBM20* familial DCM patient-specific cell lines and control showed hundreds of differential genes with 50 of them showing consistent differential expression patterns between the two disease cell lines. Gene function enrichment analysis performed on these 50 genes highlighted a vital functional group of pattern specification process including *TBX18*, *CYP26B1*, *HHIP*, and *LHX2* (p ≤ 2.8E-8) which regulates cell response to differentiation during heart development.

## Conclusions

This study highlights developmental defects linked to the causative etiology of *RBM20* familial DCM due to dysfunctional cardiac gene expression in cardiogenesis. Insights gained from using patient-specific stem cells enables the anticipation of disease outcomes and targeting molecular therapy at the root cause of DCM.
